# Structure-based optimization and discovery of novel 1,3,5-triazine derivatives as bacterial translation inhibitor with favourable metabolic fate

**DOI:** 10.1186/2047-2994-4-S1-I2

**Published:** 2015-06-16

**Authors:** UP Singh, JK Srivastava, HR Bhat

**Affiliations:** 1 Department of Pharmaceutical Sciences, Sam Higginbottom Institute of Agriculture, Technology & Sciences, Allahabad, India

## Introduction

We have recently moved into an era not just of multiple resistant bacteria but of totally resistant pathogens, which now include vancomycin-resistant *enterococci*, carbapenem-resistant *Acinetobacter baumannii*, vancomycin-resistant MRSA and, very recently, NDM-1. Thus, increased incidence of bacterial resistance to currently available antibiotics necessitates the discovery and introduction of new and effective drugs. In our earlier studies, we have discovered a potent antibacterial lead molecule from 1,3,5-triazine (first generation) and its subsequent optimization till its tenth generation results much more advanced analogue with enhanced activity and less toxicity [1].

## Objectives

Present study deals with the advancement of novel derivatives of 1,3,5-triazines to increase its efficacy and potency to make them viable drug candidate (eleventh generation).

## Methods

The synthesis of analogues was achieved by means of S_N_Ar reaction utilizing distinguished amines. These molecules were then subjected to antibacterial screening against pathogenic Gram-positive and Gram-negative micro-organisms. MetaPrint2D-React from University of Cambridge, UK was utilized for the prediction of metabolites of the compounds.

## Results

Entire set of derivatives demonstrated excellent antibacterial activity (1.56 - 25 µg ml^-1^), and in some instance found equipotent to cefixime as standard. The molecular docking study on eubacterial ribosomal decoding A site (Escherichia coli 16S rRNA A site) confirmed the stability of target compounds into the inner groove of active site by making close H-bonds with highly conserved residues, e.g. Ade38, Gua37, Ade39, and Gua40. Moreover, the most active compound 7e, in MetaPrint2D-React study was not found to be deactivated by human metabolic process, which conform the utility of designed molecules.

## Conclusion

We have discovered an another novel 1,3,5-triazine analogs as potent antibacterial agent through structure-based optimization of our defined lead.

## Disclosure of interest

None declared.
